# Block-based characterization of protease specificity from substrate sequence profile

**DOI:** 10.1186/s12859-017-1851-1

**Published:** 2017-10-03

**Authors:** Enfeng Qi, Dongyu Wang, Bo Gao, Yang Li, Guojun Li

**Affiliations:** 1 School of Mathematics, Shandong University, Jinan, 250100 China; 2 The State Key Laboratory of Microbial Technology, Shandong University, Jinan, 250100 China

**Keywords:** Protease, Block, Entropy, Site cooperation

## Abstract

**Background:**

The mechanism of action of proteases has been widely studied based on substrate specificity. Prior research has been focused on the amino acids at a single amino acid site, but rarely on combinations of amino acids around the cleavage bond.

**Results:**

We propose a novel block-based approach to reveal the potential combinations of amino acids which may regulate the action of proteases. Using the entropies of eight blocks centered at a cleavage bond, we created a distance matrix for 61 proteases to compare their specificities. After quantitative analysis, we discovered a number of prominent blocks, each of which consists of successive amino acids near a cleavage bond, intuitively characterizing the site cooperation of the substrate sequences.

**Conclusion:**

This approach will help in the discovery of specific substrate sequences which may bridge between proteases and cleavage substrate as more substrate information becomes available.

**Electronic supplementary material:**

The online version of this article (10.1186/s12859-017-1851-1) contains supplementary material, which is available to authorized users.

## Background

Proteases are a category of enzymes capable of hydrolyzing peptide bonds and irreversibly modifying functions of substrate proteins. These hydrolyzations and modifications are essential for cell growth and differentiation [1, 2]. Recognition of the target substrate of a protease depends partly on the complementation between the protease active site and the sequence surrounding the scissile bond in the substrate. Proteases have pockets that accommodate substrate residues. Substrate sequences that bind the pockets are indexed by P_4_, P_3_, P_2_, P_1_, P_1_’, P_2_’, P_3_’, P_4_’ in order from N-terminal to C-terminal following the convention of Schechter and Berger [3].

Some proteases show strict specificities on the cleavage sequences of the substrates. For example, trypsin 1 requires Lys and Arg at the P_1_ site [4], and granzyme B shows strict specificity for Asp at the P_1_ site [5]. The specificity of protease has been widely used not only in identifying the biologically relevant substrates, but also in applying protease to site-specific proteolysis [6, 7]. Proteases participate in various disease processes, exhibiting a potentially huge future application in the design of new drug targets for enzyme [8, 9] and protease inhibitors [10]. Although all the proteases function in hydrolyzing peptide bonds, almost all are linked to a particular cleavage pattern [11].

The MEROPS database is a manually curated information resource for peptidases [12]. According to MEROPS, more than 10,000 known substrates are profiled for some proteases [13], so it is necessary to develop an approach to map the abundant substrate-sequence information to specificities of proteases to highlight the enzymatic preferences, especially for specific catalytic types [14]. Integrating features of substrate sequences characteristics, PoPS [15] and PROSPER [16] are proposed to predict protease substrate cleavage sites. A well-designed approach of identifying the specificity of the protease will contribute to a better method of predicting the substrate cleavage site.

Previous analyses of protease cleavage data, such as visualized sequence logos [17], iceLogo [18], heat maps [19] and several techniques [20–22], have been focused on qualitative interpretation. Using LC-MS/MS sequencing [23], a simple and rapid multiplex substrate profiling method was presented to demonstrate the substrate specificity. Further measures include using fluorogenic substrates [4], specific labeling techniques of N-terminal [24, 25], and proteome-derived peptide libraries [26–30]. Fuchs [31] developed a method to quantify protease specificity and rank proteases with the cleavage entropy of a single position. Several quantitative measures were developed [32–35], in which the specificities of proteases were shown by the occurrences of amino acids at the binding sites. As mentioned by Schilling and Overall [19] in profiling the specificity of the MMP2, the preferred amino acid residues at different site may cooperate in the hydrolysis process, therefore, it is critically important to elucidate the hydrolyzation process by the closely cooperative relationship of successive positions on the substrate sequences.

In this study, we designed a novel approach to present the protease specificity based on blocks which are composed by successive amino acids from the substrate sequence. The essential difference between our approach and previous ones lies in that we characterize the specificity of proteases based on successive amino acids rather than a single binding site. This new approach could more reliably identify protease specificity by considering cooperation among the successive sites of the substrate peptides during the hydrolyzation process.

## Methods

### Data extraction

The dataset is composed of 61 proteases for analysis as described by Fuchs [35]. The cleavage information from all experimental sources is obtained from the MEROPS database [12] and is updated according to MEROPS 10.0. This study focuses on the protease specificity directly on the active sites, ignoring differences in allosteric sites and exosite interactions. Among the data, signal peptidase complex (XS26.001) has been deleted from the dataset since the complex contains two peptidases, and it is not possible to assign a particular cleavage to one activity due to not a single component.

### Greedy algorithm for filtering the data

First, the substrate sequence with less than two amino acids is filtered out. Then all of substrate sequences left primarily are aligned pairwise. Starting from sequences with the maximum number of similar amino acids, we remove redundant sequences by greedy algorithm [36] to make sure that there is no pair of sequences whose similarity is greater than or equal to 0.875. Therefore, there are at least two different amino acid residues between any two remaining substrate sequences.

### Construction of blocks

**Fig. 1:**
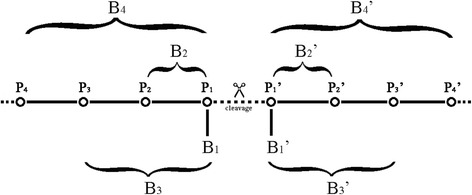
A schematic diagram of construction of different blocks. The blocks of successive amino acids are denoted from N-terminal to C-terminal, so that block B_1_ represents the P_1_ site, block B_1_’ represents the P_1_’ site, block B_2_ represents the successive sites of P_2_ and P_1_, and block B_2_’ represents the successive sites of P_1_’ and P_2_’ and so on. For example, block B_2_ LeuLys implies Leu at the site P_2_ and Lys at the site P_1_, and block B_2_’ PheArg implies Phe at the site P_1_’ and Arg at the site P_2_’. Other blocks may be deduced similarly

The indices of the residues in the substrate sequence are centered on the cleavage bond and extended to both sides incrementally, namely P_4_, P_3_, P_2_, P_1_, P_1_’, P_2_’, P_3_’, P_4_’.We define a set of eight blocks, denoted by B = (B_4_, B_3_, B_2_, B_1_, B_1_’, B_2_’, B_3_’, B_4_’), where B_1_ (B_1_’) is a vector of amino acids occurred in respective substrate sequences at P_1_ (P_1_’); B_2_ (B_2_’) is a vector of two successive amino acids occurred in respective substrate sequences at P_2_, P_1_ (P_1_’, P_2_’); B_3_ (B_3_’) is a vector of three successive amino acids occurred in respective substrate sequences at P_3_, P_2_, P_1_ (P_1_’, P_2_’, P_3_’); B_4_ (B_4_’) is a vector of four successive amino acids occurred in respective substrate sequences at P_4_, P_3_, P_2_, P_1_ (P_1_’, P_2_’, P_3_’, P_4_’). The construction of blocks is shown in Fig. 1.

### Calculation of entropy

Information entropy was firstly proposed by Shannon [37]. The block-based entropy information of the substrate reflects the specific or broad property of the protease. The randomness of the block-based substrate information is given by the entropy:

1 $$ {\mathrm{E}}_k\left(\mathrm{or}\kern0.5em {\mathrm{E}}_k^{\prime}\right)=-\sum {p}_i{\mathrm{log}}_2{p}_i\kern1em \left(k=1,\kern0.5em 2,\kern0.5em 3,\kern0.5em 4\right) $$

where *p*_i_ is the frequency of a component in block B_*k*_ (B_*k*_′). Consequently, we can get the entropy of the block B as E = (E_4_, E_3_, E_2_, E_1_, E_1_’, E_2_’, E_3_’, E_4_’).

### Calculation of distance matrix

A distance matrix is created by pairwise comparison of all 61 proteases’ cleavage bonds. The distance between two proteases is calculated by the Euclidean distance of the total entropies calculated as follows:

2 $$ d\left(P,Q\right)=\sqrt{\sum_{i=1}^4{\left[{\mathrm{E}}_i(P)-{\mathrm{E}}_i(Q)\right]}^2+\sum_{i=1}^4{\left[{\mathrm{E}}_i^{\prime }(P)-{\mathrm{E}}_i^{\prime }(Q)\right]}^2} $$

Where E_*i*_ (*P*) and E_*i*_’(*P*) are the entropies of blocks B_*i*_ and B_*i*_′ of protease *P* respectively. This yields a symmetric distance matrix. The elements on the diagonal are 0, which is the distance of identical proteases.

### Principal components analysis

All the eight blocks for each of 61 proteases are used for principal components analysis (PCA). Kaiser-Meyer-Olkin (KMO) Measure of Sampling Adequacy is computed as 0.733 which indicates that the sample size is sufficient for the application of PCA. The PCA is performed in SPSS 19.0 (SPSS Inc., Chicago, IL, USA) with the correlation method and Varimax with Kaiser Normalization as the rotation method.

### Fisher’s exact test

Fisher’s exact test [38] is used in calculating the *p*-value of combinations. We simulated the substrates according to the frequencies of the amino acids, and repeated 1000 times for the prominent combinations in each block. As the false positive would be a waste of time, the Bonferroni correction [39] is used for the *p*-value threshold by *p* < 0.05/N, where N is the number of different kinds of combinations. For one combination occurring in the input substrate sequences, we consider the number of sequences containing this combination in both experiment and background sequences. We make the null hypothesis that there’s no difference between proportions of sequences containing this combination in experiment and background sequences. The combination with significance level *p* < 0.05/N, occurring more than half of 1000 times, is regarded as a prominent combination. If a combination is significant, then the null hypothesis is rejected. The data might look like Table 1. The probability of obtaining any such set of values if given by the hypergeometric distribution:

3 $$ p=\frac{\left(\begin{array}{c}\kern1.00em a+b\kern1.00em \\ {}\kern1.00em a\kern1.00em \end{array}\right)\kern0.5em \left(\begin{array}{c}\kern1.00em c+d\kern1.00em \\ {}\kern1.00em c\kern1.00em \end{array}\right)}{\left(\begin{array}{c}\kern1.00em n\kern1.00em \\ {}\kern1.00em a+c\kern1.00em \end{array}\right)}=\frac{\left(a,+,b\right)!\kern0.5em \left(c,+,d\right)!\kern0.5em \left(a,+,c\right)!\kern0.5em \left(b,+,d\right)!}{a!\kern1.50em b!\kern0.5em c!\kern0.5em d!\kern0.5em n!} $$

where $$ \left(\begin{array}{c}\hfill n\hfill \\ {}\hfill k\hfill \end{array}\right) $$ is the binomial coefficient and the symbol ! indicates the factorial operator. The software package of methods can be obtained in Additional file 1.

**Table 1:** 2 × 2 contingency table for Fisher’s exact test

	Yes	No	Row total
Experiment data	*a*	*b*	*a* + *b*
Background data	*c*	*d*	*c* + *d*
Column total	*a* + *c*	*b* + *d*	*a* + *b* + *c* + *d*

### Creation of sequence profile

To depict the substrate preferences at different sites, the data of substrate sequences after removing the redundancy is submitted to Weblogo [17, 40] to generate sequence profiles of substrate cleavage site.

## Results

### Distance character of 61 proteases

The entropies of eight blocks B_4_, B_3_, B_2_, B_1_, B_1_’, B_2_’, B_3_’, B_4_’ are calculated and denoted as E_4_, E_3_, E_2_, E_1_, E_1_’, E_2_’, E_3_’, E_4_’ correspondingly (Additional file 2: Table S1). There are three blocks with entropy 0, including, caspase 6 with E_1_ = 0 implying the unique amino acid Asp at site P_1_; peptidyl-Lys metallopeptidase with E_1_’ = 0 implying the unique amino acid Lys at site P_1_’; lysyl peptidase with E_1_ = 0 implying the unique amino acid Lys at site P_1_.

**Fig. 2:**
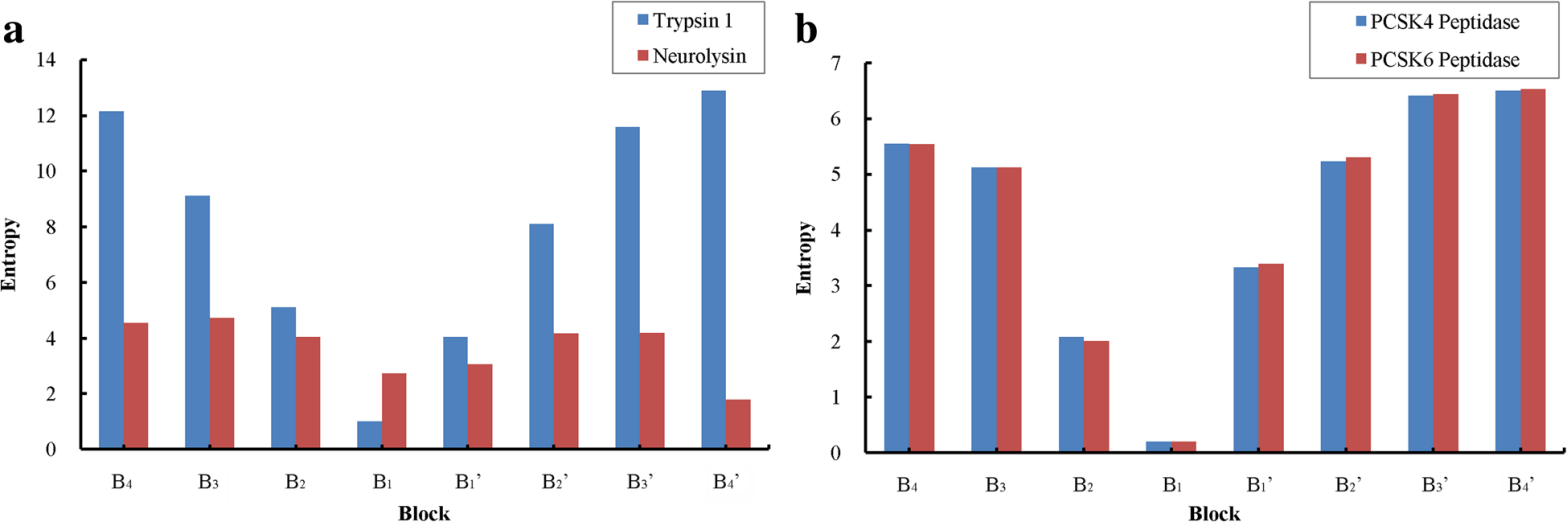
Comparisons of eight entropies of proteases with maximum and minimum distance. **a** Entropy distributions of eight blocks from proteases neurolysin and trypsin 1 with the maximum distance in the distance matrix. **b** Entropy distributions of eight blocks from proteases PCSK4 and PCSK6 with the minimum distance in the distance matrix

We obtained a distance matrix (Additional file 2: Table S2) by calculating the distances of the entropies of eight blocks between 61 proteases. We found obvious distinctions between proteases. The maximum entry 16.630 in the matrix is the distance between the proteases neurolysin and trypsin 1, with their corresponding entropies being shown in Fig. 2a. From that, all of the block entropies except B_1_ of trypsin 1 are higher than the corresponding block entropies of neurolysin. The fundamental difference between neurolysin and trypsin 1 is their different activities. Where neurolysin is an oligopeptidase unable to cleave proteins [41], trypsin 1 is an endopeptidase [4]. Another factor is the great gap in the numbers of distinct substrate sequences between neurolysin (45) and trypsin 1 (9014). Excluding the diagonal entries, the minimum entry 0.125 in the matrix is the distance between the proteases PCSK4 and PCSK6, with their corresponding entropies being shown in Fig. 2b. This is due to a large amount of similar blocks between them.

### Principal components analysis

The entropies of eight blocks reveal the complexity of different combination types. In order to mine the blocks which play the crucial role in the specificity recognition of substrate sequence, we used principal components analysis.

The distribution of eight different eigenvalues is shown in a scree plot (Additional file 2: Figure S1.). Three principal components (PC1: the first principal component; PC2: the second principal component; PC3: the third principal component) are obtained according to the principle of eigenvalues more than 1. Among the three principal components, PC1, PC2 and PC3 contribute 57.938%, 23.284% and 15.960% to the total variance respectively, and the cumulative contribution of three principal components is 97.183% (Additional file 2: Table S3). Thus, the three principal components may represent the main features in the recognition of substrate specificities of different proteases.

**Fig. 3:**
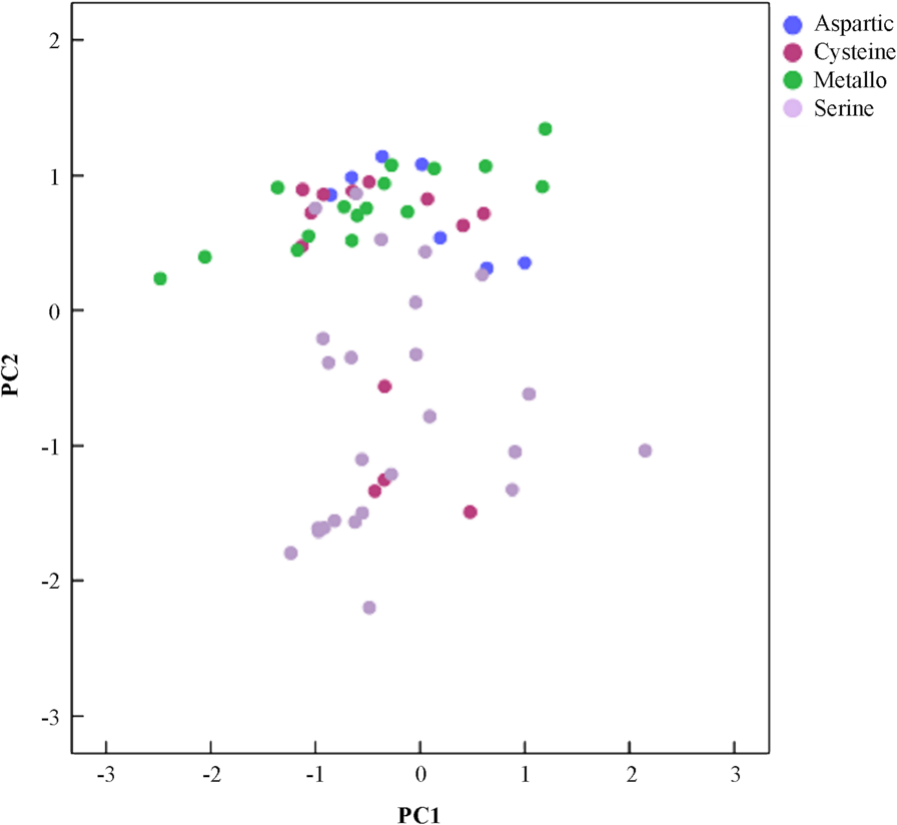
Scatter plot of principal component analysis from PC1versus PC2. The selected data is grouped into four types according to the MEROPS database, including aspartic, cysteine, metallo and serine. Coloring according to catalytic types, aspartic protease: blue; cysteine protease: red; metallo protease: green; serine protease: pink

PC1 shows a strongly positive correlation with E_4_, E_3_, E_2_’, E_3_’, E_4_’ demonstrated by the principal components load matrix (Additional file 2: Table S4). The lower the entropies are, the more prominent blocks there will be at the corresponding binding sites. As E_1_, E_2_ and E_1_’ possess a weak correlation with the composition of PC1, the corresponding B_1_, B_2_ and B_1_’ are most likely to have the prominent blocks. PC2 correlates with E_1_ and E_2_ (Additional file 2: Table S4). From the scatter plot of PC2 versus PC1 in Fig. 3, note that PC2 separates metallo proteases from serine proteases approximately. Almost all of the proteases from metallo and aspartic proteases are above the zero of the vertical axis implying a positive correlation with PC2.

### Block-based sequence profile

**Fig. 4:**
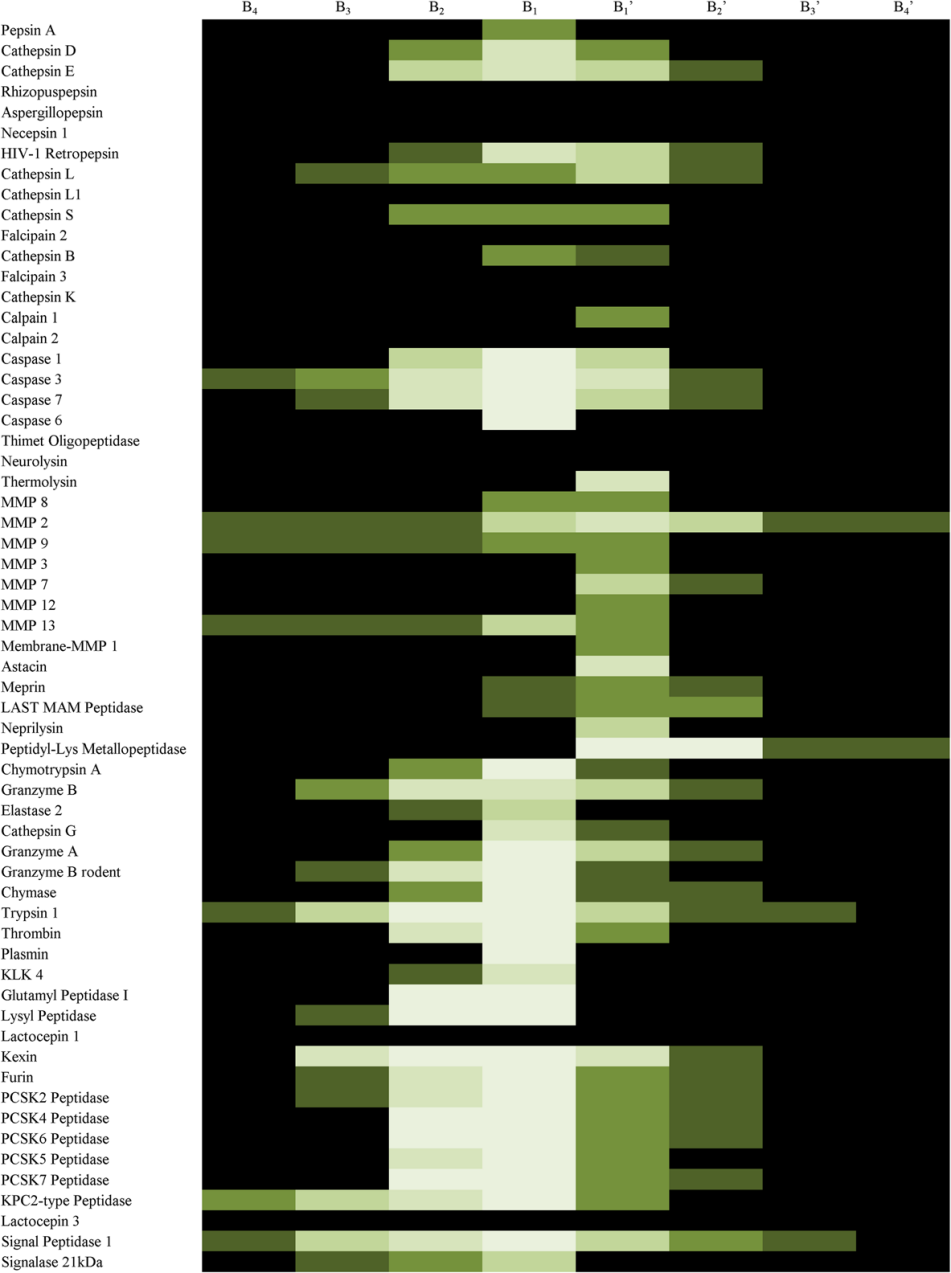
Heat map of prominent combinations in each block. Five shades are shown ranging from darkest green (less 20% of substrates) to brightest green (greater 80% of substrates), and black background indicates no prominent combination in the block

**Fig. 5:**
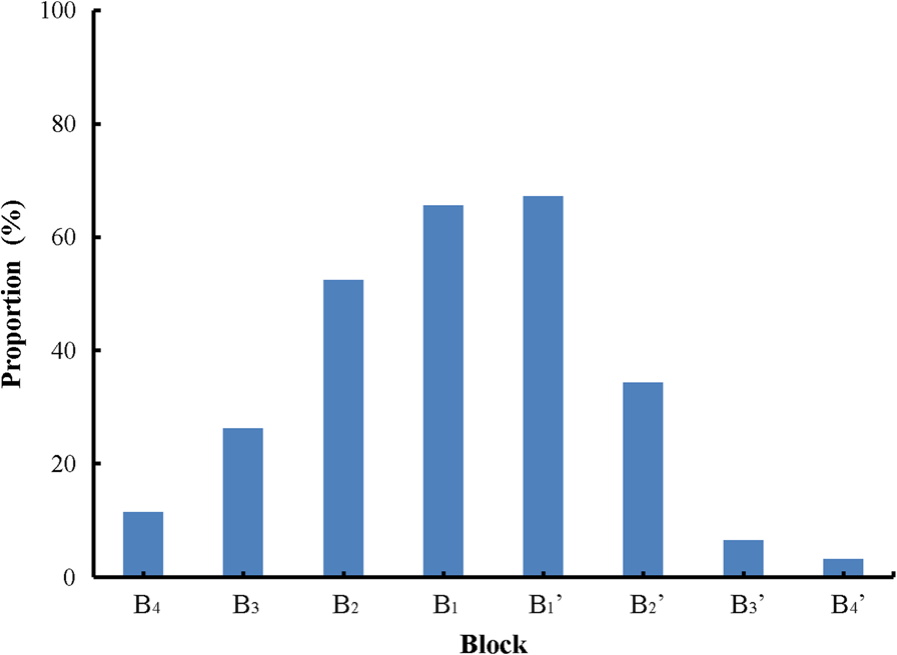
The proportion of proteases with prominent blocks. The horizontal axis shows eight blocks B_4_, B_3_, B_2_, B_1_, B_1_’, B_2_’, B_3_’ and B_4_’. The vertical axis shows the proportions of proteases with significant block in 61 proteases. The proportions from B_4_ to B_4_’ are 11.475%, 26.230%, 52.459%, 65.574%, 67.213%, 34.426%, 6.557% and 3.279% respectively

Our algorithm has uncovered a number of prominent blocks in different proteases. The proportions of prominent combinations in the substrate at each block are presented by different shades of green in the heat map (Fig. 4), indicating that a large number of significant combinations can be analyzed with this approach. For each block, the proportions of proteases possessing a prominent block in 61 proteases is demonstrated in Fig. 5, the ratios at B_2_, and B_2_’ are higher than those at B_4_, B_3_, B_3_’ and B_4_’, implying that the amino acids close to the cleavage bond cooperate more preferably than those far away.

There are a few prominent blocks from prime side. For instance, except for strict specificity for Lys at the P_1_’ site, peptidyl-Lys metallopeptidase has block B_2_’ with LysGlu = 179 from 1869 substrates, and signal peptidase 1 has block B_3_’ with AlaGluAla = 19 from 297 substrates (The number behind the equal sign represents the amount of combination of amino acids in the corresponding block).

Meanwhile, a few blocks from non-prime side show the specificity. For example, kexin has block B_2_ LysArg = 147 from 171 substrates. With caspase 3 having 571 substrates, besides the prominent block B_3_ GluValAsp = 43, we still find the prominent block B_4_ AspGluValAsp = 19.

**Fig. 6:**
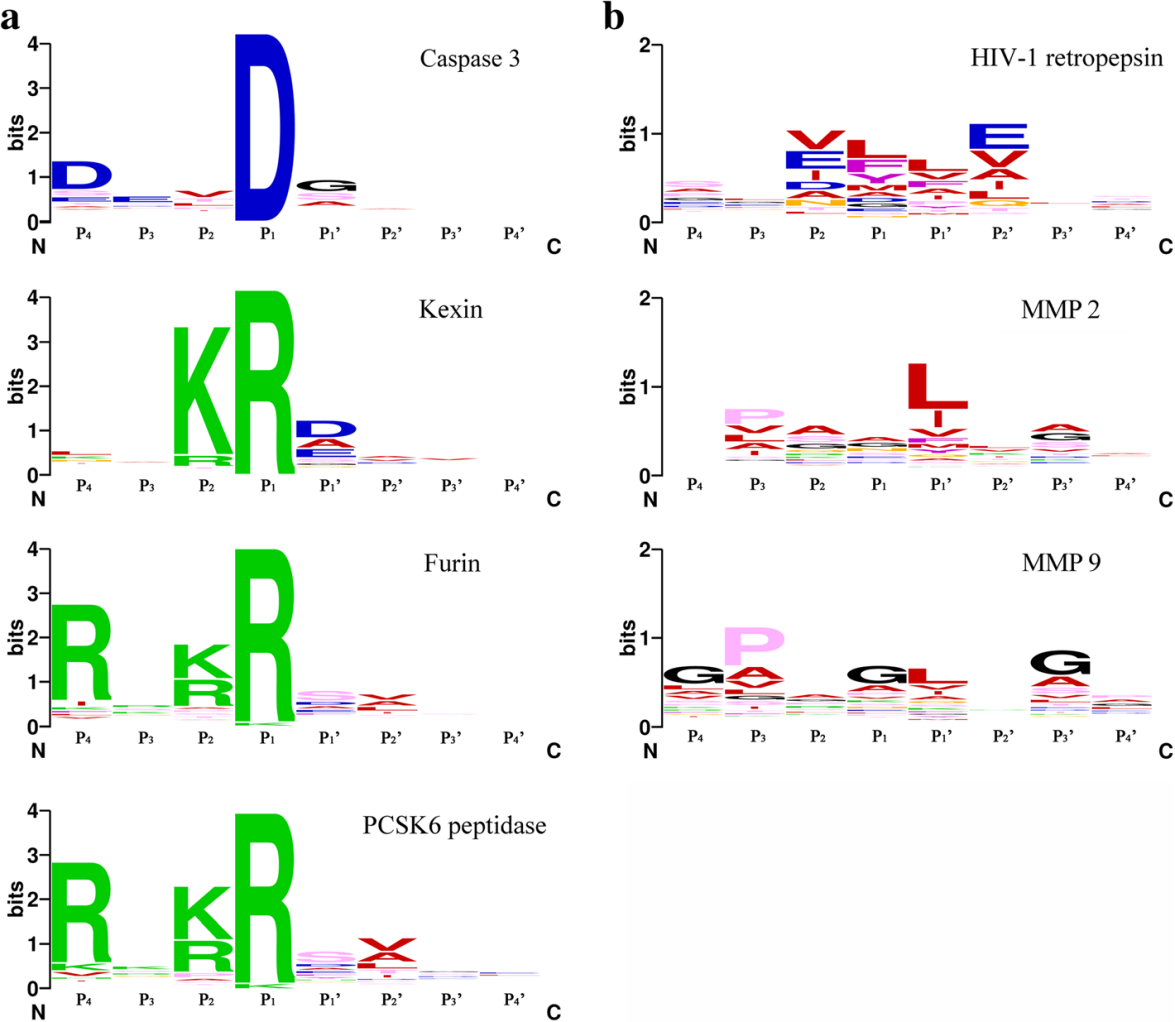
Cleavage site sequence logos and the prominent B_2_ blocks of proteases. **a** The sequence logos of caspase 3, kexin, furin and PCSK6 peptidase, which have obvious specificity at the site P_1_. **b** The sequence logos of HIV-1 retropepsin, MMP 2 and MMP 9, which have multiple preferences at sites P_1_ and P_2_

Some proteases show the specificity at site P_1_, and the prominent B_2_ blocks (Table 2) are apparent in the sequence logos shown in Fig. 6a. For example, B_2_ block ValAsp in caspase 3, and B_2_ block LysArg in kexin, furin and PCSK6 peptidase. However, in the sequence logos shown in Fig. 6b, there are two or more amino acids in the binding sites P_1_ and P_2_, which indicates the preference rather than the strict specificity. As listed in Table 2, the top three amino acids of HIV-1 retropepsin at sites P_2_ and P_1_ are Val, Glu, Ile and Leu, Phe, Tyr, respectively, yet the prominent block B_2_ with the highest number of combination are GluLeu = 35. For MMP2, the top three amino acids at sites P_2_ and P_1_ are Ala, Ser, Gly and Ala, Gly, Asn, respectively, and the prominent block B_2_ with the highest number of combination is AlaAla = 100. For MMP 9, amino acids on the top at sites P_2_ and P_1_ are all unpolar amino acids such as Ala, Gly, Pro and Gly, Ala, Pro respectively, yet the top one Gly at the site P_1_ has the preference of Pro at the site P_2_ forming the prominent block B_2_ ProGly = 25, and Pro at the site P_2_ shows no preference of the top amino acids at the site P_1_ except Gly in the formed block B_2_.

**Table 2:** The top prominent B_2_ blocks of proteases listed in Fig. 6

Protease	P_2_	P_1_	Block B_2_
(a) The top prominent B_2_ blocks of proteases listed in Fig. 6a			
Caspase 3	Val	Asp	ValAsp
Kexin	Lys	Arg	LysArg
Furin	Lys, Arg	Arg	LysArg, ArgArg
PCSK6 peptidase	Lys, Arg	Arg	LysArg, ArgArg
(b) The top prominent B_2_ blocks of proteases listed in Fig. 6b			
HIV-1 Retropepsin	Val, Glu, Ile	Leu, Phe,Tyr	GluLeu, ValLeu
MMP2	Ala, Ser, Gly	Ala, Gly, Asn	AlaAla, SerGly
MMP9	Ala, Gly, Pro	Gly, Ala, Pro	ProGly, AlaAla

Some blocks B_2_’ show the similar combination property as in blocks B_2_. For example, the top three amino acids of HIV-1 retropepsin at sites P_1_’ and P_2_’ are Leu, Val, Phe and Glu, Val, Ala, respectively, yet the prominent block B_2_’ with the highest number of combination are LeuAla = 33. For LAST_MAM peptidase, amino acids on the top at sites P_1_’ and P_2_’ are Asp, Ala, Glu and Pro, Ala, Glu respectively, yet the top one Asp at the site P_1_’ shows no preference of the top amino acid Pro at the site P_2_’, and the prominent block B_2_’ with the highest number of combination is AlaPro = 44 from 429 substrates. For the proteases which cleavage sites possess two or more preferred residues, the prominent combinations in the blocks reflect the cooperation of the residues in one position with other positions, characterizing the specificity of proteases detailedly.

## Discussion

Some specificities of certain proteases have been determined, such as trypsin 1 [4], caspase 3 [42], kexin [43], furin [44] and so on. However, by focusing on single positions and not taking into consideration the interaction of adjacent amino acids, the study of substrate specificity is too limited.

Taking the cooperation of amino acids into account, we propose a quantitative method to characterize substrates specificity of different proteases. By calculating entropies of different blocks, some distinctions of substrates between different proteases can be conceived (Fig. 2a). The principal component analysis gives evidence on the existence of blocks which play the crucial role in the specificity recognition of substrate sequence, and most of them are block B_2_, B_1_ and B_1_’. This is confirmed by the statistical analysis showing the ratios at B_2_, B_1_, and B_1_’ are higher than those in other blocks (Fig. 5). With Fisher’s exact test, a number of prominent blocks of different proteases have been discovered. For example, blocks B_2_ in kexin and furin are consistent with the previous discovery that both of the proteases cleave after dibasic residues [45]. Other block B_2_, e.g. GluLeu in HIV-1 retropepsin, AlaAla in MMP2 and ProGly in MMP 9, are more likely to reflect the preferences and the cooperation of the successive amino acids in the substrate sequences which could not be found previously.

Cathepsin B is an endopeptidase and as an exopeptidase acts as a peptidyl-dipeptidase, releasing a dipeptide from the C-terminus of a protein or peptide. As no distinction is made in MEROPS between cleavages resulting from either activity, a view of the endopeptidase activity would be clear if the substrates of the exopeptidase activity were filtered out.

From the specificity matrix in MEROPS and the heat map [19], the preference of the protease is shown by the amino acids at one single binding site. However, it will not show the combinations of amino acids if proteases show multiple preferences at each binding site. Our method indicates interactions of different compositions of successive amino acids which can’t be obtained previously. For example, MMP9 has preferences for Ala, Gly and Pro at the site P_2_, Gly, Ala and Pro at the site P_1_ from the specificity matrix, yet the combination is clear using our method, such as ProGly, AlaAla in block B_2_. Whether a prominent combination exists in a block is obviously presented in the heat map of prominent combinations in each block (Fig. 4). These findings of specific blocks will shed light on future experiments and further investigation of proteolytic specificity.

Although in this study we only focused on the specificity of selected proteases, the method would be applicable to other proteases for mining the specificity pattern of substrates. In conclusion, we can obtain more substrate specificity patterns by site cooperation as more and more substrates data becomes available. Further investigations of the substrate specificity will be important to reveal the hydrolyzation mechanism of proteases.

## Conclusions

Generally, the design of experiments and the description of the specificity of the protease are based on the assumption that the process of binding amino acid residue to the corresponding subsite is independent. However, it is not exactly true and the binding of amino acid residues at one site can more or less influence the binding at other subsites. It is essential to take the site cooperation into consideration for understanding fully the active site.

Our approach provides a new framework for dealing with the specificity pattern of substrates of the proteases. The combinations of site cooperation in the substrates offer a new sight in mining the specificity of the protease. We successfully find the significant blocks B_2_ in kexin and furin which are consistent with the previous discovery that both of the proteases cleave after dibasic residues. Other significant combinations found by the new approach could be more reliable to capture the activity of the active site. In principle, this method is useful for the further research relying on the substrate dataset, such as the identification of the novel substrate and the design of the inhibitor for the protease.

## Additional files

Additional file 1: Software package. A .tar.gz file that contains Perl and C++ scripts and an example to illustrate our approach. The package also includes a manual file (txt) for the instruction of the software. (GZ 11 kb)

Additional file 2: Supplementary Information. A .pdf file including Supplementary Tables and Figures. (PDF 65 kb)
