# Supplementary material for: Block-based characterization of protease specificity from substrate sequence profile
Source: BMC Bioinformatics. 2017 Oct 3;18:438. doi: 10.1186/s12859-017-1851-1 (PMC5627433; doi:10.1186/s12859-017-1851-1)
Supplement: Supplementary file 2 — Supplementary Information. A .pdf file including Supplementary Tables and Figures. (PDF 65 kb) [file 12859_2017_1851_MOESM2_ESM.pdf]

Supplementary Information

Block-based characterization of protease specificity from substrate sequence profile

Enfeng Qi[1], Dongyu Wang[2], Bo Gao[1], Yang Li[1], Guojun Li[1,*]

[1]School of Mathematics, Shandong University, Jinan 250100, China

[2]The State Key Laboratory of Microbial Technology, Shandong University, Jinan 250100, China

[*]Corresponding authors: G. Li

Figure legend

Figure S1. Distribution of principal components.

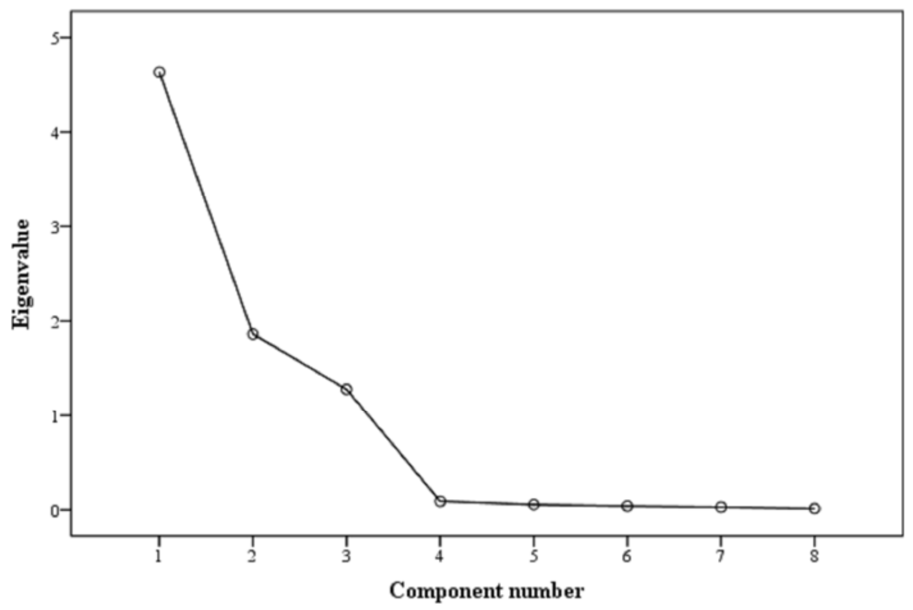

Figure S1. Distribution of principal components. The horizontal axis represents the principal components, and the vertical axis represents eigenvalues. The component 1, component 2 and component 3 are chosen for the subsequent analysis for their eigenvalues more than 1.

Table legends

Table S1. The entropies of eight blocks of 61 proteases.

Table S2. The distance matrix of 61 proteases.

Table S3. The Eigenvalues and corresponding contributions of the principal components.

Table S4. Principal components load matrix.

Table S1. The entropies of eight blocks of 61 proteases.

	Protease	MEROPS[a]	Catalytic Type	Number[b]	E_4	E_3	E_2	E_1	E_1’	E_2’	E_3’	E_4’
1	Pepsin A	A01.001	Aspartic	387	8.467	8.384	7.288	3.893	4.058	7.387	8.317	8.238
2	Cathepsin D	A01.009	Aspartic	727	9.407	8.759	6.352	2.706	3.767	7.206	9.134	9.443
3	Cathepsin E	A01.010	Aspartic	1287	10.218	9.346	6.517	2.802	3.516	7.043	9.543	10.214
4	Rhizopuspepsin	A01.012	Aspartic	240	7.850	7.826	7.100	4.113	4.088	6.986	7.701	7.712
5	Aspergillopepsin	A01.016	Aspartic	156	7.233	7.237	6.648	3.976	4.031	6.731	7.225	7.201
6	Necepsin 1	A01.053	Aspartic	117	6.853	6.830	6.417	3.732	3.903	6.383	6.802	6.853
7	HIV-1 Retropepsin	A02.001	Aspartic	632	8.280	8.306	6.290	3.350	3.582	6.371	8.586	9.108
8	Cathepsin L	C01.032	Cysteine	1065	8.536	9.056	6.569	3.809	3.646	7.301	9.446	9.938
9	Cathepsin L1	C01.033	Cysteine	192	7.483	7.452	6.689	3.913	4.006	6.835	7.477	7.539
10	Cathepsin S	C01.034	Cysteine	732	8.236	8.729	6.346	3.636	3.661	7.211	9.078	9.464
11	Falcipain 2	C01.046	Cysteine	137	6.656	6.797	6.254	3.842	3.919	6.276	6.753	6.784
12	Cathepsin B	C01.060	Cysteine	589	8.372	8.460	6.804	3.511	3.783	6.928	8.080	8.644
13	Falcipain 3	C01.063	Cysteine	114	6.319	6.710	6.113	3.896	3.856	5.924	6.403	6.365
14	Cathepsin K	C01.036	Cysteine	121	5.254	6.117	5.575	3.427	3.683	6.190	6.671	6.704
15	Calpain 1	C02.001	Cysteine	100	6.476	6.396	5.785	3.827	3.510	5.941	6.440	6.421
16	Calpain 2	C02.002	Cysteine	156	7.242	7.140	6.329	3.957	3.709	6.437	7.114	7.191
17	Caspase 1	C14.001	Cysteine	170	7.247	6.694	4.606	1.265	3.173	6.115	7.252	7.327
18	Caspase 3	C14.003	Cysteine	571	8.171	6.825	3.621	0.037	3.353	6.857	8.819	9.129
19	Caspase 7	C14.004	Cysteine	149	6.585	5.802	3.541	0.058	2.820	5.746	7.021	7.206
20	Caspase 6	C14.005	Cysteine	189	7.178	5.789	4.057	0.000	3.871	6.619	7.425	7.541
21	Thimet Oligopeptidase	M03.001	Metallo	46	4.665	4.863	4.648	3.321	3.137	4.735	5.081	3.644
22	Neurolysin	M03.002	Metallo	44	4.535	4.726	4.042	2.730	3.062	4.172	4.195	1.778
23	Thermolysin	M04.001	Metallo	260	7.697	7.740	7.063	4.067	2.951	6.299	7.593	7.675
24	MMP 8	M10.002	Metallo	104	6.332	6.275	5.683	3.395	3.407	5.804	6.284	6.381
25	MMP 2	M10.003	Metallo	2502	10.404	9.781	7.432	3.863	3.037	6.865	9.655	11.021
26	MMP 9	M10.004	Metallo	340	7.868	7.637	6.651	3.572	3.636	6.897	8.017	8.290
27	MMP 3	M10.005	Metallo	171	7.213	7.033	6.209	3.582	3.682	6.434	7.162	7.292
28	MMP 7	M10.008	Metallo	174	7.317	7.112	6.297	3.705	2.542	5.586	6.983	7.222
29	MMP 12	M10.009	Metallo	171	7.071	6.645	5.897	3.390	3.482	6.257	6.972	7.304
30	MMP 13	M10.013	Metallo	138	5.610	6.194	5.647	3.039	3.408	6.062	6.536	5.663
31	Membrane-MMP 1	M10.014	Metallo	127	6.957	6.863	6.343	3.632	3.453	6.145	6.957	6.973
32	Astacin	M12.001	Metallo	206	7.634	7.570	6.763	3.891	2.791	5.830	7.354	7.657
33	Meprin	M12.002	Metallo	766	9.496	9.297	7.556	3.867	3.555	7.102	9.122	9.522
34	LAST MAM Peptidase	M12.033	Metallo	429	8.726	8.545	7.236	3.898	3.130	6.179	8.113	8.641
35	Neprilysin	M13.001	Metallo	102	5.953	6.238	6.001	3.872	2.976	5.610	5.756	5.190
36	Peptidyl-Lys Metallopeptidase	M35.004	Metallo	1869	10.823	10.464	8.117	4.123	0.000	4.174	8.125	10.456
37	Chymotrypsin A	S01.001	Serine	711	9.274	8.758	6.287	2.536	3.995	7.573	9.086	9.247
38	Granzyme B	S01.010	Serine	1568	9.884	8.182	5.119	1.525	3.727	7.362	9.795	10.475
39	Elastase 2	S01.131	Serine	364	7.936	8.147	6.298	2.860	4.015	7.223	8.354	8.465
40	Cathepsin G	S01.133	Serine	244	7.034	7.526	6.303	3.021	3.944	6.919	7.707	7.795
41	Granzyme A	S01.135	Serine	286	8.036	7.494	5.150	1.520	3.536	6.329	7.814	7.978
42	Granzyme B rodent	S01.136	Serine	341	8.158	7.182	4.411	0.969	3.698	6.641	8.022	8.329
43	Chymase	S01.140	Serine	100	6.624	6.389	4.875	1.716	3.908	5.737	6.448	6.449
44	Trypsin 1	S01.151	Serine	9014	12.162	9.111	5.095	0.996	4.054	8.116	11.601	12.897
45	Thrombin	S01.217	Serine	136	6.512	6.301	3.317	0.570	3.255	6.047	6.791	6.928
46	Plasmin	S01.233	Serine	121	6.797	6.718	5.031	1.240	3.761	6.199	6.774	6.791
47	KLK 4	S01.251	Serine	112	6.390	6.297	4.537	1.534	3.833	6.001	6.513	6.591
48	Glutamyl Peptidase I	S01.269	Serine	1045	9.115	8.017	4.586	0.549	3.992	7.731	9.675	9.981
49	Lysyl Peptidase	S01.280	Serine	778	9.371	7.851	4.112	0.000	4.071	7.869	9.431	9.580
50	Lactocepin 1	S08.019	Serine	101	6.638	6.619	5.980	3.737	3.960	6.106	6.619	6.611
51	Kexin	S08.070	Serine	171	6.737	4.516	0.896	0.052	3.011	5.863	7.063	7.359
52	Furin	S08.071	Serine	172	6.012	5.494	2.540	0.211	3.491	5.855	7.083	7.312
53	PCSK2 Peptidase	S08.073	Serine	166	6.659	5.628	2.393	0.457	3.734	6.214	7.108	7.221
54	PCSK4 Peptidase	S08.074	Serine	94	5.551	5.122	2.083	0.204	3.335	5.234	6.419	6.504
55	PCSK6 Peptidase	S08.075	Serine	94	5.543	5.121	2.009	0.204	3.391	5.304	6.448	6.533
56	PCSK5 Peptidase	S08.076	Serine	116	5.904	5.389	2.333	0.216	3.546	5.583	6.724	6.789
57	PCSK7 Peptidase	S08.077	Serine	102	5.619	5.172	2.099	0.239	3.333	5.351	6.547	6.594
58	KPC2-type Peptidase	S08.109	Serine	104	4.384	3.758	1.616	0.534	3.079	5.318	6.359	6.566
59	Lactocepin 3	S08.116	Serine	158	7.291	7.266	6.364	3.853	4.031	6.743	7.240	7.291
60	Signal Peptidase 1	S26.001	Serine	297	7.261	5.297	4.037	0.518	2.970	5.788	7.538	7.882
61	Signalase 21kDa	S26.010	Serine	316	8.081	7.136	5.808	2.398	3.672	6.946	8.034	8.186

[a]The index of 61 proteases in the MEROPS database. [b]The number of substrate sequences which have been removed redundancy.

Table S2. The distance matrix of 61 proteases[a].

	1	2	3	4	5	6	7	8	9	10	11	12	13	14	15	16	17
1	0.000	2.356	3.406	1.263	2.441	3.324	1.850	2.308	1.923	1.844	3.562	0.951	4.261	5.025	4.404	2.747	4.777
2	2.356	0.000	1.375	3.322	4.209	4.914	1.743	1.574	3.681	1.501	5.133	1.960	5.814	6.322	5.815	4.316	4.861
3	3.406	1.375	0.000	4.468	5.438	6.158	2.790	2.024	4.903	2.421	6.375	3.077	7.040	7.594	7.037	5.526	6.037
4	1.263	3.322	4.468	0.000	1.228	2.131	2.240	3.265	0.770	2.638	2.360	1.495	3.049	3.916	3.229	1.588	4.234
5	2.441	4.209	5.438	1.228	0.000	0.928	2.929	4.237	0.552	3.517	1.147	2.439	1.861	2.755	2.041	0.561	3.599
6	3.324	4.914	6.158	2.131	0.928	0.000	3.571	5.024	1.426	4.275	0.313	3.212	1.015	1.999	1.186	0.747	3.252
7	1.850	1.743	2.790	2.240	2.929	3.571	0.000	1.794	2.434	1.161	3.747	1.069	4.392	4.895	4.405	2.945	4.005
8	2.308	1.574	2.024	3.265	4.237	5.024	1.794	0.000	3.700	0.804	5.203	2.056	5.883	6.321	5.934	4.379	5.553
9	1.923	3.681	4.903	0.770	0.552	1.426	2.434	3.700	0.000	2.987	1.650	1.904	2.372	3.178	2.515	0.888	3.646
10	1.844	1.501	2.421	2.638	3.517	4.275	1.161	0.804	2.987	0.000	4.451	1.442	5.132	5.551	5.172	3.640	4.808
11	3.562	5.133	6.375	2.360	1.147	0.313	3.747	5.203	1.650	4.451	0.000	3.434	0.755	1.771	0.961	0.920	3.294
12	0.951	1.960	3.077	1.495	2.439	3.212	1.069	2.056	1.904	1.442	3.434	0.000	4.114	4.800	4.204	2.596	4.209
13	4.261	5.814	7.040	3.049	1.861	1.015	4.392	5.883	2.372	5.132	0.755	4.114	0.000	1.511	0.601	1.600	3.494
14	5.025	6.322	7.594	3.916	2.755	1.999	4.895	6.321	3.178	5.551	1.771	4.800	1.511	0.000	1.415	2.519	3.303
15	4.404	5.815	7.037	3.229	2.041	1.186	4.405	5.934	2.515	5.172	0.961	4.204	0.601	1.415	0.000	1.669	3.203
16	2.747	4.316	5.526	1.588	0.561	0.747	2.945	4.379	0.888	3.640	0.920	2.596	1.600	2.519	1.669	0.000	3.293
17	4.777	4.861	6.037	4.234	3.599	3.252	4.005	5.553	3.646	4.808	3.294	4.209	3.494	3.303	3.203	3.293	0.000
18	5.715	4.511	5.324	5.800	5.698	5.743	4.545	5.419	5.482	4.941	5.843	5.088	6.257	5.947	5.996	5.534	3.104
19	6.778	6.643	7.729	6.219	5.474	4.966	5.881	7.355	5.587	6.645	4.942	6.193	4.944	4.322	4.591	5.145	2.039
20	5.992	5.769	6.909	5.574	4.982	4.649	5.264	6.610	5.017	5.921	4.693	5.494	4.890	4.399	4.616	4.788	1.882
21	8.580	9.875	11.101	7.413	6.220	5.340	8.523	10.100	6.726	9.324	5.118	8.402	4.439	4.135	4.282	5.915	5.868
22	10.526	11.756	12.962	9.399	8.247	7.390	10.508	12.109	8.746	11.331	7.190	10.357	6.511	6.336	6.383	7.954	7.591
23	2.083	3.648	4.686	1.346	1.537	2.070	2.284	3.577	1.313	2.951	2.262	1.901	2.825	3.683	2.886	1.476	3.938
24	4.728	6.013	7.242	3.589	2.412	1.519	4.624	6.206	2.860	5.432	1.332	4.468	0.926	1.296	0.537	2.037	2.949
25	4.072	2.821	1.765	5.139	6.219	6.991	3.688	2.558	5.685	3.211	7.201	3.885	7.854	8.486	7.886	6.323	7.365
26	1.392	2.676	3.889	1.085	1.651	2.375	1.439	2.730	1.139	2.008	2.596	1.054	3.311	3.893	3.347	1.770	3.622
27	2.818	4.207	5.449	1.746	0.783	0.782	2.857	4.359	1.008	3.597	0.972	2.582	1.655	2.398	1.656	0.425	2.903
28	3.509	4.666	5.732	2.591	1.951	1.723	3.210	4.792	2.095	4.096	1.782	3.129	2.035	2.807	1.870	1.476	3.124
29	3.385	4.565	5.799	2.360	1.373	0.946	3.204	4.772	1.626	4.002	1.019	3.065	1.526	2.045	1.335	0.948	2.532
30	5.326	6.640	7.919	4.222	3.046	2.205	5.320	6.823	3.508	6.039	2.021	5.147	1.566	1.217	1.429	2.787	3.241
31	3.304	4.746	5.960	2.178	1.109	0.571	3.346	4.874	1.482	4.124	0.678	3.092	1.201	2.073	1.190	0.698	3.004
32	2.633	3.916	4.940	1.849	1.694	1.949	2.453	3.943	1.600	3.294	2.101	2.275	2.571	3.422	2.564	1.422	3.580
33	2.144	1.777	1.844	3.276	4.422	5.247	2.307	1.511	3.889	1.859	5.471	2.144	6.141	6.857	6.231	4.602	6.062
34	1.619	2.420	3.162	1.988	2.876	3.564	1.461	2.395	2.420	2.023	3.774	1.207	4.373	5.203	4.441	2.917	4.684
35	5.724	7.242	8.437	4.546	3.412	2.583	5.861	7.377	3.916	6.628	2.396	5.593	1.744	2.202	1.652	3.108	4.210
36	6.509	5.955	5.347	7.011	7.725	8.176	5.895	5.899	7.373	6.131	8.337	6.108	8.722	9.522	8.691	7.594	8.479
37	2.299	0.528	1.737	3.267	4.128	4.837	1.934	1.771	3.608	1.607	5.060	1.989	5.749	6.224	5.762	4.273	4.761
38	4.434	2.237	2.309	5.213	5.838	6.375	3.410	3.212	5.368	3.247	6.564	3.954	7.209	7.409	7.092	5.854	5.267
39	1.571	2.048	3.384	1.839	2.410	3.068	1.328	2.381	1.931	1.637	3.275	1.103	3.965	4.390	4.017	2.564	3.527
40	2.307	3.482	4.793	1.618	1.335	1.741	2.281	3.626	1.132	2.847	1.923	2.011	2.593	2.952	2.673	1.440	2.894
41	3.591	3.324	4.495	3.390	3.217	3.281	2.692	4.172	3.031	3.475	3.421	2.946	3.873	4.053	3.718	3.039	1.595
42	4.377	3.694	4.774	4.288	4.116	4.155	3.391	4.630	3.930	3.999	4.271	3.750	4.701	4.652	4.491	3.951	1.829
43	5.233	5.907	7.153	4.362	3.394	2.720	4.822	6.458	3.668	5.672	2.662	4.818	2.556	2.396	2.347	3.099	1.691
44	7.773	5.581	4.691	8.795	9.624	10.257	7.029	6.121	9.113	6.575	10.459	7.484	11.131	11.373	11.036	9.696	9.222
45	6.439	6.451	7.593	5.831	5.042	4.533	5.648	7.113	5.181	6.374	4.485	5.881	4.465	3.892	4.143	4.723	1.795
46	4.866	5.339	6.590	4.161	3.360	2.865	4.413	5.978	3.525	5.204	2.886	4.415	2.977	2.802	2.801	3.127	1.120
47	5.454	6.025	7.279	4.625	3.670	3.028	4.982	6.562	3.921	5.775	2.962	5.019	2.883	2.466	2.641	3.383	1.583
48	4.904	3.053	3.545	5.502	5.907	6.315	3.937	4.042	5.506	3.862	6.485	4.412	7.081	7.072	6.953	5.919	4.686
49	5.443	3.716	4.242	5.952	6.247	6.574	4.560	4.830	5.894	4.584	6.737	4.961	7.290	7.260	7.134	6.235	4.627
50	3.922	5.390	6.635	2.738	1.534	0.675	4.014	5.522	2.019	4.760	0.444	3.756	0.553	1.589	0.648	1.249	3.148
51	8.907	8.542	9.516	8.374	7.615	7.127	7.918	9.217	7.732	8.561	7.051	8.338	7.010	6.260	6.579	7.263	4.513
52	7.452	7.322	8.423	6.857	6.056	5.538	6.537	7.928	6.193	7.219	5.463	6.888	5.424	4.605	5.084	5.746	2.923
53	7.115	6.937	8.039	6.564	5.812	5.357	6.258	7.616	5.925	6.911	5.300	6.574	5.319	4.650	4.970	5.515	2.718
54	8.463	8.525	9.637	7.733	6.800	6.145	7.617	9.102	7.025	8.369	6.023	7.921	5.810	4.980	5.464	6.449	3.875
55	8.478	8.529	9.642	7.754	6.825	6.179	7.632	9.104	7.047	8.372	6.057	7.937	5.852	5.014	5.506	6.479	3.903
56	7.860	7.844	8.960	7.191	6.318	5.728	7.008	8.458	6.505	7.731	5.630	7.316	5.500	4.709	5.162	5.991	3.282
57	8.320	8.357	9.468	7.607	6.690	6.054	7.466	8.934	6.904	8.204	5.937	7.777	5.747	4.912	5.396	6.342	3.735
58	9.665	9.870	11.002	8.853	7.839	7.135	8.857	10.283	8.110	9.554	6.978	9.174	6.709	5.623	6.338	7.493	5.321
59	2.442	4.065	5.313	1.299	0.330	0.964	2.788	4.140	0.555	3.397	1.167	2.353	1.892	2.715	2.017	0.503	3.355
60	6.118	5.863	6.917	5.638	5.012	4.620	5.149	6.605	5.054	5.930	4.642	5.540	4.796	4.283	4.414	4.701	1.837
61	2.562	2.767	4.068	2.378	2.394	2.724	2.011	3.415	2.093	2.680	2.924	2.073	3.561	3.843	3.443	2.346	2.431

	18	19	20	21	22	23	24	25	26	27	28	29	30	31	32	33
1	5.715	6.778	5.992	8.580	10.526	2.083	4.728	4.072	1.392	2.818	3.509	3.385	5.326	3.304	2.633	2.144
2	4.511	6.643	5.769	9.875	11.756	3.648	6.013	2.821	2.676	4.207	4.666	4.565	6.640	4.746	3.916	1.777
3	5.324	7.729	6.909	11.101	12.962	4.686	7.242	1.765	3.889	5.449	5.732	5.799	7.919	5.960	4.940	1.844
4	5.800	6.219	5.574	7.413	9.399	1.346	3.589	5.139	1.085	1.746	2.591	2.360	4.222	2.178	1.849	3.276
5	5.698	5.474	4.982	6.220	8.247	1.537	2.412	6.219	1.651	0.783	1.951	1.373	3.046	1.109	1.694	4.422
6	5.743	4.966	4.649	5.340	7.390	2.070	1.519	6.991	2.375	0.782	1.723	0.946	2.205	0.571	1.949	5.247
7	4.545	5.881	5.264	8.523	10.508	2.284	4.624	3.688	1.439	2.857	3.210	3.204	5.320	3.346	2.453	2.307
8	5.419	7.355	6.610	10.100	12.109	3.577	6.206	2.558	2.730	4.359	4.792	4.772	6.823	4.874	3.943	1.511
9	5.482	5.587	5.017	6.726	8.746	1.313	2.860	5.685	1.139	1.008	2.095	1.626	3.508	1.482	1.600	3.889
10	4.941	6.645	5.921	9.324	11.331	2.951	5.432	3.211	2.008	3.597	4.096	4.002	6.039	4.124	3.294	1.859
11	5.843	4.942	4.693	5.118	7.190	2.262	1.332	7.201	2.596	0.972	1.782	1.019	2.021	0.678	2.101	5.471
12	5.088	6.193	5.494	8.402	10.357	1.901	4.468	3.885	1.054	2.582	3.129	3.065	5.147	3.092	2.275	2.144
13	6.257	4.944	4.890	4.439	6.511	2.825	0.926	7.854	3.311	1.655	2.035	1.526	1.566	1.201	2.571	6.141
14	5.947	4.322	4.399	4.135	6.336	3.683	1.296	8.486	3.893	2.398	2.807	2.045	1.217	2.073	3.422	6.857
15	5.996	4.591	4.616	4.282	6.383	2.886	0.537	7.886	3.347	1.656	1.870	1.335	1.429	1.190	2.564	6.231
16	5.534	5.145	4.788	5.915	7.954	1.476	2.037	6.323	1.770	0.425	1.476	0.948	2.787	0.698	1.422	4.602
17	3.104	2.039	1.882	5.868	7.591	3.938	2.949	7.365	3.622	2.903	3.124	2.532	3.241	3.004	3.580	6.062
18	0.000	3.467	2.653	8.751	10.382	5.765	5.857	6.876	4.884	5.161	5.542	4.979	6.160	5.517	5.591	6.194
19	3.467	0.000	1.663	5.832	7.263	5.860	4.193	9.102	5.585	4.764	4.823	4.280	4.182	4.744	5.443	7.967
20	2.653	1.663	0.000	6.582	8.116	5.536	4.318	8.397	4.942	4.383	4.863	4.021	4.386	4.534	5.259	7.212
21	8.751	5.832	6.582	0.000	2.312	6.993	4.000	12.045	7.560	5.876	5.697	5.471	3.431	5.377	6.598	10.412
22	10.382	7.263	8.116	2.312	0.000	8.992	6.091	13.975	9.585	7.914	7.692	7.535	5.501	7.430	8.592	12.338
23	5.765	5.860	5.536	6.993	8.992	0.000	3.195	5.240	1.357	1.607	1.585	2.054	3.904	1.789	0.677	3.564
24	5.857	4.193	4.318	4.000	6.091	3.195	0.000	8.144	3.617	1.934	2.034	1.505	1.141	1.467	2.818	6.520
25	6.876	9.102	8.397	12.045	13.975	5.240	8.144	0.000	4.715	6.328	6.437	6.717	8.885	6.783	5.546	1.983
26	4.884	5.585	4.942	7.560	9.585	1.357	3.617	4.715	0.000	1.713	2.419	2.173	4.280	2.233	1.694	3.017
27	5.161	4.764	4.383	5.876	7.914	1.607	1.934	6.328	1.713	0.000	1.448	0.643	2.673	0.629	1.483	4.638
28	5.542	4.823	4.863	5.697	7.692	1.585	2.034	6.437	2.419	1.448	0.000	1.371	2.869	1.185	1.005	4.932
29	4.979	4.280	4.021	5.471	7.535	2.054	1.505	6.717	2.173	0.643	1.371	0.000	2.335	0.664	1.763	5.114
30	6.160	4.182	4.386	3.431	5.501	3.904	1.141	8.885	4.280	2.673	2.869	2.335	0.000	2.236	3.612	7.193
31	5.517	4.744	4.534	5.377	7.430	1.789	1.467	6.783	2.233	0.629	1.185	0.664	2.236	0.000	1.539	5.100
32	5.591	5.443	5.259	6.598	8.592	0.677	2.818	5.546	1.694	1.483	1.005	1.763	3.612	1.539	0.000	3.985
33	6.194	7.967	7.212	10.412	12.338	3.564	6.520	1.983	3.017	4.638	4.932	5.114	7.193	5.100	3.985	0.000
34	5.700	6.637	6.141	8.563	10.498	1.737	4.714	3.593	1.707	2.970	2.991	3.409	5.473	3.325	2.037	2.022
35	7.227	5.186	5.533	3.053	5.060	4.061	1.548	9.231	4.791	3.150	2.927	2.878	1.471	2.590	3.729	7.549
36	8.824	9.968	9.928	12.157	13.825	6.289	8.856	4.507	6.672	7.638	6.873	7.865	9.662	7.785	6.276	5.156
37	4.395	6.542	5.586	9.795	11.660	3.726	5.955	3.208	2.661	4.152	4.742	4.521	6.530	4.712	4.022	2.053
38	3.521	6.559	5.595	10.855	12.681	5.444	7.190	3.832	4.366	5.639	6.077	5.803	7.770	6.174	5.555	3.778
39	4.263	5.455	4.631	8.083	10.031	2.335	4.214	4.513	1.136	2.413	3.232	2.815	4.750	2.977	2.607	2.878
40	4.611	4.801	4.178	6.714	8.727	1.885	2.853	5.785	1.253	1.206	2.306	1.586	3.343	1.703	1.998	4.081
41	2.766	3.432	2.773	7.123	8.890	3.287	3.657	5.893	2.642	2.682	3.091	2.623	4.149	3.038	3.084	4.623
42	1.750	3.131	2.230	7.597	9.299	4.295	4.389	6.302	3.487	3.583	4.064	3.449	4.798	3.956	4.108	5.237
43	4.620	2.639	2.685	4.486	6.238	4.164	1.964	8.378	4.120	2.790	3.124	2.360	2.096	2.659	3.775	6.863
44	6.948	10.289	9.293	14.841	16.630	9.109	11.164	5.209	8.087	9.531	9.924	9.741	11.771	10.078	9.293	6.345
45	3.609	0.989	1.750	5.502	6.958	5.576	3.775	8.984	5.289	4.366	4.567	3.913	3.747	4.373	5.185	7.751
46	3.948	2.414	2.161	5.269	6.976	4.012	2.484	7.888	3.788	2.759	3.194	2.417	2.603	2.767	3.691	6.407
47	4.389	2.277	2.363	4.589	6.330	4.449	2.257	8.541	4.312	3.055	3.434	2.605	2.272	2.968	4.079	7.063
48	2.478	5.719	4.644	10.412	12.155	5.778	6.972	5.158	4.640	5.634	6.206	5.724	7.387	6.150	5.844	4.766
49	2.204	5.461	4.342	10.356	11.984	6.228	7.111	5.912	5.129	5.929	6.510	5.967	7.478	6.422	6.251	5.466
50	5.810	4.701	4.520	4.759	6.824	2.607	0.990	7.511	2.921	1.235	1.896	1.082	1.746	0.899	2.370	5.808
51	4.705	2.958	3.645	6.943	8.014	8.127	6.251	10.934	7.731	6.932	7.066	6.418	6.240	6.963	7.715	10.006
52	3.869	1.388	2.166	5.931	7.305	6.640	4.716	9.826	6.259	5.387	5.625	4.899	4.623	5.399	6.246	8.702
53	3.540	1.605	1.913	6.107	7.481	6.437	4.652	9.493	5.981	5.165	5.501	4.710	4.644	5.241	6.075	8.365
54	5.239	2.250	3.366	5.235	6.317	7.431	5.044	10.996	7.246	6.131	6.220	5.599	4.828	6.022	6.978	9.814
55	5.216	2.285	3.360	5.304	6.386	7.468	5.093	11.007	7.265	6.161	6.271	5.632	4.876	6.061	7.021	9.828
56	4.519	1.714	2.654	5.504	6.730	6.954	4.767	10.357	6.670	5.654	5.842	5.149	4.609	5.608	6.532	9.182
57	5.046	2.112	3.205	5.317	6.446	7.316	4.986	10.834	7.105	6.020	6.129	5.492	4.781	5.927	6.871	9.660
58	6.564	3.749	4.753	5.365	6.372	8.528	5.936	12.270	8.384	7.205	7.281	6.634	5.610	7.040	8.087	11.090
59	5.422	5.248	4.744	6.221	8.242	1.599	2.365	6.157	1.554	0.618	1.918	1.216	3.025	1.094	1.700	4.391
60	2.838	1.386	1.467	6.348	7.982	5.312	4.102	8.259	4.907	4.306	4.420	3.834	4.322	4.369	4.923	7.202
61	3.475	4.335	3.519	7.318	9.246	2.504	3.537	5.316	1.549	2.037	2.774	2.162	4.110	2.543	2.509	3.859

	34	35	36	37	38	39	40	41	42	43	44	45	46	47	48
1	1.619	5.724	6.509	2.299	4.434	1.571	2.307	3.591	4.377	5.233	7.773	6.439	4.866	5.454	4.904
2	2.420	7.242	5.955	0.528	2.237	2.048	3.482	3.324	3.694	5.907	5.581	6.451	5.339	6.025	3.053
3	3.162	8.437	5.347	1.737	2.309	3.384	4.793	4.495	4.774	7.153	4.691	7.593	6.590	7.279	3.545
4	1.988	4.546	7.011	3.267	5.213	1.839	1.618	3.390	4.288	4.362	8.795	5.831	4.161	4.625	5.502
5	2.876	3.412	7.725	4.128	5.838	2.410	1.335	3.217	4.116	3.394	9.624	5.042	3.360	3.670	5.907
6	3.564	2.583	8.176	4.837	6.375	3.068	1.741	3.281	4.155	2.720	10.257	4.533	2.865	3.028	6.315
7	1.461	5.861	5.895	1.934	3.410	1.328	2.281	2.692	3.391	4.822	7.029	5.648	4.413	4.982	3.937
8	2.395	7.377	5.899	1.771	3.212	2.381	3.626	4.172	4.630	6.458	6.121	7.113	5.978	6.562	4.042
9	2.420	3.916	7.373	3.608	5.368	1.931	1.132	3.031	3.930	3.668	9.113	5.181	3.525	3.921	5.506
10	2.023	6.628	6.131	1.607	3.247	1.637	2.847	3.475	3.999	5.672	6.575	6.374	5.204	5.775	3.862
11	3.774	2.396	8.337	5.060	6.564	3.275	1.923	3.421	4.271	2.662	10.459	4.485	2.886	2.962	6.485
12	1.207	5.593	6.108	1.989	3.954	1.103	2.011	2.946	3.750	4.818	7.484	5.881	4.415	5.019	4.412
13	4.373	1.744	8.722	5.749	7.209	3.965	2.593	3.873	4.701	2.556	11.131	4.465	2.977	2.883	7.081
14	5.203	2.202	9.522	6.224	7.409	4.390	2.952	4.053	4.652	2.396	11.373	3.892	2.802	2.466	7.072
15	4.441	1.652	8.691	5.762	7.092	4.017	2.673	3.718	4.491	2.347	11.036	4.143	2.801	2.641	6.953
16	2.917	3.108	7.594	4.273	5.854	2.564	1.440	3.039	3.951	3.099	9.696	4.723	3.127	3.383	5.919
17	4.684	4.210	8.479	4.761	5.267	3.527	2.894	1.595	1.829	1.691	9.222	1.795	1.120	1.583	4.686
18	5.700	7.227	8.824	4.395	3.521	4.263	4.611	2.766	1.750	4.620	6.948	3.609	3.948	4.389	2.478
19	6.637	5.186	9.968	6.542	6.559	5.455	4.801	3.432	3.131	2.639	10.289	0.989	2.414	2.277	5.719
20	6.141	5.533	9.928	5.586	5.595	4.631	4.178	2.773	2.230	2.685	9.293	1.750	2.161	2.363	4.644
21	8.563	3.053	12.157	9.795	10.855	8.083	6.714	7.123	7.597	4.486	14.841	5.502	5.269	4.589	10.412
22	10.498	5.060	13.825	11.660	12.681	10.031	8.727	8.890	9.299	6.238	16.630	6.958	6.976	6.330	12.155
23	1.737	4.061	6.289	3.726	5.444	2.335	1.885	3.287	4.295	4.164	9.109	5.576	4.012	4.449	5.778
24	4.714	1.548	8.856	5.955	7.190	4.214	2.853	3.657	4.389	1.964	11.164	3.775	2.484	2.257	6.972
25	3.593	9.231	4.507	3.208	3.832	4.513	5.785	5.893	6.302	8.378	5.209	8.984	7.888	8.541	5.158
26	1.707	4.791	6.672	2.661	4.366	1.136	1.253	2.642	3.487	4.120	8.087	5.289	3.788	4.312	4.640
27	2.970	3.150	7.638	4.152	5.639	2.413	1.206	2.682	3.583	2.790	9.531	4.366	2.759	3.055	5.634
28	2.991	2.927	6.873	4.742	6.077	3.232	2.306	3.091	4.064	3.124	9.924	4.567	3.194	3.434	6.206
29	3.409	2.878	7.865	4.521	5.803	2.815	1.586	2.623	3.449	2.360	9.741	3.913	2.417	2.605	5.724
30	5.473	1.471	9.662	6.530	7.770	4.750	3.343	4.149	4.798	2.096	11.771	3.747	2.603	2.272	7.387
31	3.325	2.590	7.785	4.712	6.174	2.977	1.703	3.038	3.956	2.659	10.078	4.373	2.767	2.968	6.150
32	2.037	3.729	6.276	4.022	5.555	2.607	1.998	3.084	4.108	3.775	9.293	5.185	3.691	4.079	5.844
33	2.022	7.549	5.156	2.053	3.778	2.878	4.081	4.623	5.237	6.863	6.345	7.751	6.407	7.063	4.766
34	0.000	5.688	5.101	2.666	4.419	2.168	2.763	3.507	4.403	5.271	7.747	6.409	4.960	5.538	5.104
35	5.688	0.000	9.506	7.189	8.567	5.464	4.110	5.001	5.770	3.056	12.526	4.774	3.658	3.351	8.370
36	5.101	9.506	0.000	6.410	6.976	6.931	7.606	7.453	8.115	9.334	8.518	9.995	9.032	9.596	8.085
37	2.666	7.189	6.410	0.000	2.284	1.881	3.348	3.232	3.555	5.785	5.676	6.314	5.184	5.883	2.886
38	4.419	8.567	6.976	2.284	0.000	3.631	4.897	3.893	3.614	6.636	4.018	6.529	5.985	6.621	1.524
39	2.168	5.464	6.931	1.881	3.631	0.000	1.481	2.260	2.948	4.251	7.396	5.131	3.740	4.371	3.739
40	2.763	4.110	7.606	3.348	4.897	1.481	0.000	2.269	3.115	3.163	8.776	4.419	2.817	3.315	4.791
41	3.507	5.001	7.453	3.232	3.893	2.260	2.269	0.000	1.120	2.833	7.869	3.212	2.189	2.887	3.515
42	4.403	5.770	8.115	3.555	3.614	2.948	3.115	1.120	0.000	3.261	7.497	2.978	2.579	3.158	2.926
43	5.271	3.056	9.334	5.785	6.636	4.251	3.163	2.833	3.261	0.000	10.605	2.152	0.921	0.557	6.098
44	7.747	12.526	8.518	5.676	4.018	7.396	8.776	7.869	7.497	10.605	0.000	10.337	9.946	10.584	4.828
45	6.409	4.774	9.995	6.314	6.529	5.131	4.419	3.212	2.978	2.152	10.337	0.000	1.985	1.721	5.688
46	4.960	3.658	9.032	5.184	5.985	3.740	2.817	2.189	2.579	0.921	9.946	1.985	0.000	0.909	5.361
47	5.538	3.351	9.596	5.883	6.621	4.371	3.315	2.887	3.158	0.557	10.584	1.721	0.909	0.000	5.990
48	5.104	8.370	8.085	2.886	1.524	3.739	4.791	3.515	2.926	6.098	4.828	5.688	5.361	5.990	0.000
49	5.676	8.481	8.641	3.510	2.241	4.235	5.156	3.626	2.855	6.041	5.207	5.434	5.294	5.903	0.930
50	4.089	2.148	8.572	5.315	6.728	3.549	2.201	3.442	4.246	2.356	10.650	4.227	2.697	2.672	6.598
51	8.834	7.122	11.976	8.439	7.955	7.579	7.057	5.679	5.026	4.915	11.247	3.118	4.944	4.510	7.066
52	7.442	5.704	10.965	7.190	7.092	6.081	5.404	4.223	3.756	3.199	10.728	1.400	3.152	2.741	6.158
53	7.174	5.696	10.795	6.786	6.680	5.762	5.181	3.904	3.353	3.101	10.302	1.337	3.013	2.663	5.757
54	8.389	5.714	11.784	8.411	8.424	7.189	6.346	5.320	5.006	3.664	12.078	2.227	3.897	3.276	7.539
55	8.419	5.776	11.835	8.410	8.410	7.196	6.363	5.336	5.003	3.707	12.054	2.242	3.930	3.310	7.515
56	7.837	5.601	11.342	7.714	7.704	6.545	5.780	4.667	4.288	3.253	11.360	1.620	3.365	2.829	6.792
57	8.257	5.698	11.674	8.240	8.237	7.033	6.212	5.168	4.832	3.591	11.888	2.081	3.791	3.188	7.346
58	9.633	6.430	13.062	9.762	9.735	8.445	7.485	6.790	6.479	4.985	13.335	3.844	5.312	4.590	8.870
59	2.858	3.463	7.713	3.978	5.628	2.244	1.158	2.957	3.835	3.220	9.447	4.798	3.161	3.476	5.667
60	5.953	5.307	9.350	5.802	5.680	4.831	4.323	2.894	2.529	2.804	9.390	1.926	2.513	2.560	4.994
61	2.790	4.897	7.296	2.652	3.755	1.369	1.490	1.351	2.030	3.327	7.712	4.100	2.806	3.421	3.660

	49	50	51	52	53	54	55	56	57	58	59	60	61
1	5.443	3.922	8.907	7.452	7.115	8.463	8.478	7.860	8.320	9.665	2.442	6.118	2.562
2	3.716	5.390	8.542	7.322	6.937	8.525	8.529	7.844	8.357	9.870	4.065	5.863	2.767
3	4.242	6.635	9.516	8.423	8.039	9.637	9.642	8.960	9.468	11.002	5.313	6.917	4.068
4	5.952	2.738	8.374	6.857	6.564	7.733	7.754	7.191	7.607	8.853	1.299	5.638	2.378
5	6.247	1.534	7.615	6.056	5.812	6.800	6.825	6.318	6.690	7.839	0.330	5.012	2.394
6	6.574	0.675	7.127	5.538	5.357	6.145	6.179	5.728	6.054	7.135	0.964	4.620	2.724
7	4.560	4.014	7.918	6.537	6.258	7.617	7.632	7.008	7.466	8.857	2.788	5.149	2.011
8	4.830	5.522	9.217	7.928	7.616	9.102	9.104	8.458	8.934	10.283	4.140	6.605	3.415
9	5.894	2.019	7.732	6.193	5.925	7.025	7.047	6.505	6.904	8.110	0.555	5.054	2.093
10	4.584	4.760	8.561	7.219	6.911	8.369	8.372	7.731	8.204	9.554	3.397	5.930	2.680
11	6.737	0.444	7.051	5.463	5.300	6.023	6.057	5.630	5.937	6.978	1.167	4.642	2.924
12	4.961	3.756	8.338	6.888	6.574	7.921	7.937	7.316	7.777	9.174	2.353	5.540	2.073
13	7.290	0.553	7.010	5.424	5.319	5.810	5.852	5.500	5.747	6.709	1.892	4.796	3.561
14	7.260	1.589	6.260	4.605	4.650	4.980	5.014	4.709	4.912	5.623	2.715	4.283	3.843
15	7.134	0.648	6.579	5.084	4.970	5.464	5.506	5.162	5.396	6.338	2.017	4.414	3.443
16	6.235	1.249	7.263	5.746	5.515	6.449	6.479	5.991	6.342	7.493	0.503	4.701	2.346
17	4.627	3.148	4.513	2.923	2.718	3.875	3.903	3.282	3.735	5.321	3.355	1.837	2.431
18	2.204	5.810	4.705	3.869	3.540	5.239	5.216	4.519	5.046	6.564	5.422	2.838	3.475
19	5.461	4.701	2.958	1.388	1.605	2.250	2.285	1.714	2.112	3.749	5.248	1.386	4.335
20	4.342	4.520	3.645	2.166	1.913	3.366	3.360	2.654	3.205	4.753	4.744	1.467	3.519
21	10.356	4.759	6.943	5.931	6.107	5.235	5.304	5.504	5.317	5.365	6.221	6.348	7.318
22	11.984	6.824	8.014	7.305	7.481	6.317	6.386	6.730	6.446	6.372	8.242	7.982	9.246
23	6.228	2.607	8.127	6.640	6.437	7.431	7.468	6.954	7.316	8.528	1.599	5.312	2.504
24	7.111	0.990	6.251	4.716	4.652	5.044	5.093	4.767	4.986	5.936	2.365	4.102	3.537
25	5.912	7.511	10.934	9.826	9.493	10.996	11.007	10.357	10.834	12.270	6.157	8.259	5.316
26	5.129	2.921	7.731	6.259	5.981	7.246	7.265	6.670	7.105	8.384	1.554	4.907	1.549
27	5.929	1.235	6.932	5.387	5.165	6.131	6.161	5.654	6.020	7.205	0.618	4.306	2.037
28	6.510	1.896	7.066	5.625	5.501	6.220	6.271	5.842	6.129	7.281	1.918	4.420	2.774
29	5.967	1.082	6.418	4.899	4.710	5.599	5.632	5.149	5.492	6.634	1.216	3.834	2.162
30	7.478	1.746	6.240	4.623	4.644	4.828	4.876	4.609	4.781	5.610	3.025	4.322	4.110
31	6.422	0.899	6.963	5.399	5.241	6.022	6.061	5.608	5.927	7.040	1.094	4.369	2.543
32	6.251	2.370	7.715	6.246	6.075	6.978	7.021	6.532	6.871	8.087	1.700	4.923	2.509
33	5.466	5.808	10.006	8.702	8.365	9.814	9.828	9.182	9.660	11.090	4.391	7.202	3.859
34	5.676	4.089	8.834	7.442	7.174	8.389	8.419	7.837	8.257	9.633	2.858	5.953	2.790
35	8.481	2.148	7.122	5.704	5.696	5.714	5.776	5.601	5.698	6.430	3.463	5.307	4.897
36	8.641	8.572	11.976	10.965	10.795	11.784	11.835	11.342	11.674	13.062	7.713	9.350	7.296
37	3.510	5.315	8.439	7.190	6.786	8.411	8.410	7.714	8.240	9.762	3.978	5.802	2.652
38	2.241	6.728	7.955	7.092	6.680	8.424	8.410	7.704	8.237	9.735	5.628	5.680	3.755
39	4.235	3.549	7.579	6.081	5.762	7.189	7.196	6.545	7.033	8.445	2.244	4.831	1.369
40	5.156	2.201	7.057	5.404	5.181	6.346	6.363	5.780	6.212	7.485	1.158	4.323	1.490
41	3.626	3.442	5.679	4.223	3.904	5.320	5.336	4.667	5.168	6.790	2.957	2.894	1.351
42	2.855	4.246	5.026	3.756	3.353	5.006	5.003	4.288	4.832	6.479	3.835	2.529	2.030
43	6.041	2.356	4.915	3.199	3.101	3.664	3.707	3.253	3.591	4.985	3.220	2.804	3.327
44	5.207	10.650	11.247	10.728	10.302	12.078	12.054	11.360	11.888	13.335	9.447	9.390	7.712
45	5.434	4.227	3.118	1.400	1.337	2.227	2.242	1.620	2.081	3.844	4.798	1.926	4.100
46	5.294	2.697	4.944	3.152	3.013	3.897	3.930	3.365	3.791	5.312	3.161	2.513	2.806
47	5.903	2.672	4.510	2.741	2.663	3.276	3.310	2.829	3.188	4.590	3.476	2.560	3.421
48	0.930	6.598	7.066	6.158	5.757	7.539	7.515	6.792	7.346	8.870	5.667	4.994	3.660
49	0.000	6.804	6.641	5.866	5.411	7.227	7.198	6.475	7.035	8.612	5.992	4.823	3.964
50	6.804	0.000	6.751	5.188	5.028	5.683	5.718	5.314	5.604	6.636	1.506	4.451	3.073
51	6.641	6.751	0.000	2.108	2.078	2.204	2.138	2.086	2.093	2.880	7.354	3.387	6.445
52	5.866	5.188	2.108	0.000	0.846	1.437	1.402	0.739	1.240	2.857	5.812	2.178	5.033
53	5.411	5.028	2.078	0.846	0.000	1.938	1.889	1.208	1.743	3.392	5.548	2.137	4.696
54	7.227	5.683	2.204	1.437	1.938	0.000	0.125	0.774	0.217	1.906	6.583	3.235	6.134
55	7.198	5.718	2.138	1.402	1.889	0.125	0.000	0.742	0.192	1.890	6.606	3.258	6.146
56	6.475	5.314	2.086	0.739	1.208	0.774	0.742	0.000	0.593	2.461	6.086	2.661	5.493
57	7.035	5.604	2.093	1.240	1.743	0.217	0.192	0.593	0.000	1.987	6.468	3.084	5.979
58	8.612	6.636	2.880	2.857	3.392	1.906	1.890	2.461	1.987	0.000	7.653	4.457	7.387
59	5.992	1.506	7.354	5.812	5.548	6.583	6.606	6.086	6.468	7.653	0.000	4.785	2.161
60	4.823	4.451	3.387	2.178	2.137	3.235	3.258	2.661	3.084	4.457	4.785	0.000	3.591
61	3.964	3.073	6.445	5.033	4.696	6.134	6.146	5.493	5.979	7.387	2.161	3.591	0.000

[a]The 61 preteases are represented by the corresponding index 1 - 61 in the first column of Table S1.

Table S3. The Eigenvalues and corresponding contributions of the principal components.

Component	Total	% of Variance	Cumulative %
1	4.635	57.938	57.938
2	1.863	23.284	81.223
3	1.277	15.960	97.183
4	0.090	1.124	98.307
5	0.055	0.682	99.989
6	0.040	0.494	99.483
7	0.028	0.351	99.834
8	0.013	0.166	100.000

Table S4. Principal components load matrix[a].

	Component		
	1	2	3
E_4	0.955	-	-
E_3	0.803	-	-
E_2	-	0.939	-
E_1	-	0.983	-
E_1’	-	-	0.991
E_2’	0.692	-	0.690
E_3’	0.973	-	-
E_4’	0.982	-	-

[a]The coefficients < 0.6 are not shown in the load matrix for their weak influencing to entropies.
